# Dr. John Snow

**Published:** 1858-09

**Authors:** 


DEATH OF DR. JOHN SNOW.—"We announce," says the *Medical Times and Gazette*, "with great pain and grief the death of our distinguished and estimable brother, Dr. Snow. He was suddenly seized with paralysis on Thursday, 10th instant, and died on Wednesday, the 16th, at his residence, 18 Sackville Street, at 3 P.M. Drs. Murchison and Budd assiduously attended him to the last. Dr. Snow was only in his forty-sixth year, and both on public and private grounds his early loss will be very greatly regretted. His name was known to the profession chiefly in connection with chloroform, to which subject he had of late years devoted a great deal of attention, and next to Dr. Simpson he was more deservedly looked upon as the highest authority respecting the properties and administration of this agent. He had instituted at great labor a vast number of experiments for the purpose of ascertaining its effects on the lower animals, and the manner in which it might be given with the least amount of danger. His labors were fully appreciated by the profession, and for several years the leading surgeons in London constantly sought his co-operation. He was very successful in the administration of chloroform, and we believe that only one death happened in his hands from this agent. Dr. Snow's labors were not by any means confined to this subject; he is well known as having devoted great attention to the investigation of cholera, and his views regarding the propagation of the disease by drinking impure water are familiar to the profession. Dr. Snow was a man of high integrity and moral worth, possessing great abilities and untiring energy, with an unassuming disposition; and his loss will be deeply felt by many who appreciated his sterling character, and valued his friendship."—June 19, 1858.

